# Molecular mechanisms of human papilloma virus related skin cancers: A review

**DOI:** 10.1097/MD.0000000000038202

**Published:** 2024-05-24

**Authors:** Elena-Codruta Cozma, Laura Mădălina Banciu, Ana Maria Celarel, Elena Soare, Bahadar S. Srichawla, Vincent Kipkorir, Mihnea-Alexandru Găman

**Affiliations:** a University of Medicine and Pharmacy of Craiova, Craiova, Romania; b Elias University Emergency Hospital, Bucharest, Romania; c University of Massachusetts Chan Medical School, Worcester, Massachusetts, USA; d Department of Human Anatomy and Physiology, University of Nairobi, Nairobi, Kenya; e Faculty of Medicine, “Carol Davila” University of Medicine and Pharmacy, Bucharest, Romania; f Department of Hematology, Center of Hematology and Bone Marrow Transplantation, Fundeni Clinical Institute, Bucharest, Romania.

**Keywords:** Bowen disease, Bowenoid papulosis, HPV, skin cancers, squamous cell carcinoma

## Abstract

The human papillomavirus (HPV) belongs to the *Papillomaviridae* family of viruses which includes small, double-stranded DNA viral agents. Approximately 90% of HPV infections occur asymptomatically and resolve spontaneously. However, infection with high-risk viral strains can lead to the development of preneoplastic lesions, with an increased propensity to become cancerous. The location of these malignancies includes the oral cavity, cervix, vagina, anus, and vulva, among others. The role of HPV in carcinogenesis has already been demonstrated for the aforementioned neoplasia. However, regarding skin malignancies, the mechanisms that pinpoint the role played by HPV in their initiation and progression still elude our sight. Until now, the only fully understood mechanism of viral cutaneous oncogenesis is that of human herpes virus 8 infection in Kaposi sarcoma. In the case of HPV infection, however, most data focus on the role that beta strains exhibit in the oncogenesis of cutaneous squamous cell carcinoma (cSCC), along with ultraviolet radiation (UVR) and other environmental or genetic factors. However, recent epidemiological investigations have highlighted that HPV could also trigger the onset of other non-melanocytic, for example, basal cell carcinoma (BCC), and/or melanocytic skin cancers, for example, melanoma. Herein, we provide an overview of the role played by HPV in benign and malignant skin lesions with a particular focus on the main epidemiological, pathophysiological, and molecular aspects delineating the involvement of HPV in skin cancers.

## 1. Introduction

The human papillomavirus (HPV) belongs to the *Papillomaviridae* family of viruses, which includes small, double-stranded DNA viruses. More than 200 HPV genotypes have been recorded until now. Approximately 90% of HPV infections occur asymptomatically and resolve spontaneously.^[1,2]^ In some cases, cutaneous lesions such as cutaneous and genital warts may occur and persist, requiring therapeutic interventions. Moreover, infection with high-risk viral strains can lead to the appearance of preneoplastic lesions, with an increased propensity to become cancerous. The location of these malignancies includes the oral cavity, cervix, vagina, anus, and vulva among others.^[3]^ Cervical cancer is the fourth most prevalent cancer among women, and HPV is its most common cause.^[4]^ Although in the case of cancers with the locations mentioned above, the role of HPV in carcinogenesis is known, regarding skin cancers, the mechanisms by which HPV is involved in the initiation and progression of the disease are still being discussed. Until now, the only fully understood mechanism of viral cutaneous oncogenesis is that of human herpes virus 8 infection in Kaposi sarcoma.^[5]^ In the case of HPV infection, however, most data focus on the role that beta strains have in the oncogenesis of cutaneous squamous cell carcinoma (cSCC), along with ultraviolet radiation (UVR) and other environmental or genetic factors.^[6]^ However, recent studies try to explain the role of HPV in other non-melanocytic (basal cell carcinoma [BCC]) and melanocytic (melanoma) skin cancers, as a result of the epidemiological data obtained (see Section 2).

This review aims to highlight the main epidemiological, pathophysiological, and molecular aspects of benign and malignant lesions that can be caused by HPV infection or in the initiation and progression of which viral infection with these strains plays an important role.

## 2. Epidemiology of HPV-related skin lesions

HPV infection is, according to the Center for Disease Control, the most common sexually transmitted disease, with a most often self-limiting evolution. However, the same infection is responsible for the invasion of the basal epithelial cells with viral strains that can lead to the appearance of benign tumoral lesions or, on the contrary, precancerous lesions or even skin or mucosal cancers, sometimes life-threatening.^[2]^ Regarding the viral tropism influencing the epidemiology of associated pathologies, of the 5 HPV types (alpha, beta, gamma, mu, nu), alpha-HPVs are more frequently associated with anogenital lesions. In contrast, beta and gamma HPV are known for increased skin tropism.

The epidemiology of skin lesions induced by HPV infection varies significantly depending on the type of lesion.^[7]^ Thus, the most common HPV-related lesions are also some of the most common lesions in dermatology, namely viral warts, with a prevalence of approximately 10% to 33% in the general population.^[8,9]^ In the case of immunocompromised patients, it is considered that the vast majority of them (90%) have these types of lesions.^[9]^ Non-oncogenic, low-risk strains, namely 1–4,7,10,13,27–29,57,60, are most frequently blamed for their occurrence.^[8]^ Regarding genital lesions, the prevalence of their clinical manifestation is much lower, at approximately 1%. However, the high percentage of infection with high-risk strains, with oncogenic potential (19.7%–39.98% in the female population tested, respectively up to 80.3% in the male population tested) requires careful treatment and monitoring.^[10,11]^ Moreover, the presence of genital warts has been observed to be associated with an increased risk of anogenital, vulvar, vaginal, cervical, penile and even head and neck carcinomas.^[12]^

Regarding neoplastic lesions, the first association of HPV infection with malignant transformation appeared in the first half of the 20th century, by observing the transformations of Epidermodysplasia verruciformis lesions to cCSC, especially in photo-exposed areas; 90% of these lesions were positive for multiple HPV strains (see Section 5).^[13,14]^ Later, the presence of HPV strains was found in 43% of non-melanoma skin cancer biopsies, with a statistically significant difference compared to healthy skin biopsies (*P* = .02).^[15]^ Moreover, a study that evaluated the prevalence of HPV strains in 38 immunocompetent patients with cSCC, seborrheic keratoses, and BCC observed the presence of HPV in 33%, 70%, and, respectively, 21% of evaluated biopsy samples. The same study on 21 immunocompromised patients highlighted the presence of HPVs in 54%, 33%, respectively 83% of samples.^[15]^ Iftner et al observed the presence of HPV strains in 59.7% of SCC biopsies, respectively, in 27.8% of BCC biopsies, compared to only 4.7% in the healthy control group.^[16]^ Zakrzewska et al also compared the number of biopsy samples infected with HPVs in patients with cSCC and BCC, finding the presence of beta-HPV in 78% of patients with cSCC and 55% of those with BCC; all patients with cSCC, and 70% of those with BCC presented viral DNA at the level of the tumor or in the perilesional tissue.^[17]^

Regarding melanocytic cancers, although the link between melanoma and HPV infection is not clearly established, some studies show higher incidences of HPV strains in melanoma biopsies compared to healthy skin. Thus, a study carried out by Roussaki-Schulze et al on a group of 28 patients with melanoma found the presence of HPV strains (6 and 16) in 17.85% of the biopsies examined (compared to none in the control group), although the results are not statistically significant.^[18]^ Moreover, Dreau et al observed the presence of HPV strains in 58% of the examined biopsy samples (skin or lymph node biopsies) (*P* = .0002), the presence of these strains correlating with death from melanoma (*P* = .004) and survival (*P* = .001).^[19]^La Placa et al identified the presence of high-risk mucosal strains of HPV in primary melanoma biopsies (27%, *P* = .0166, using the GP-PCR technique, respectively 22%, *P* = .04 using the MY-PCR technique) and acquired melanocytic dysplastic nevi (24%, *P* = .0367 using the GP-PCR technique).^[20]^

However, the studies presented in the literature offer different results, due to the methods used in the detection of the strains (which present different specificities and sensitivities), due to the small groups of patients and high demographic reliability that do not allow an optimal comparison of the results.

## 3. Pathophysiology of HPV infection

The mechanisms by which HPV exhibits oncogenicity vary depending on the individual type. Low-risk HPV genotypes include HPV 6, 11, 42, 43, and 44. Symptomatic infection by these low-risk genotypes may present as skin lesions such as warts. However, these lesions are often benign and rarely progress to a cancerous type.^[21]^ High-risk HPV genotypes include HPV 16, 18, 31, 33, 35, 39, 45, 51, 52, 56, 58, 59, 66, 68, and 70.^[22]^ These high-risk HPV genotypes are often associated with cancerous lesions of the cervix.^[23]^ Among them, HPV 16 and 18 are the most prevalent offenders.^[24]^

### 3.1. Virion structure and function

**Figure 1.**
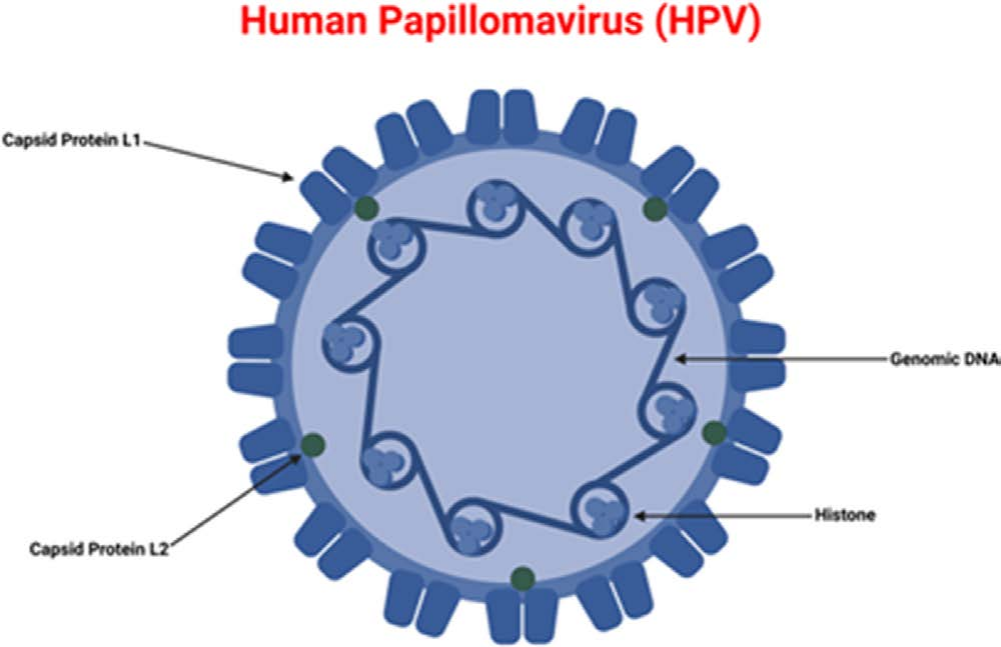
Human papillomavirus structure and major proteins. Created with BioRender.com (Agreement number: VU24TGEY28).

Structurally, HPV is a small double-stranded DNA virus that is nonenveloped. Its genome contains approximately 8kb of DNA sequences.^[25]^ This genome is functionally divided into 3 categories. The early (E) region encodes 6 proteins (E1, E2, E4, E5, E6, and E7) and their characteristics and function are described in Table 1.^[26]^ The late (L) region encodes for 2 proteins that form the viral capsid (L1 and L2).^[27]^ The long control region and late regulatory element contain cis-acting regulatory sequences that regulate viral replication, transcription, and post-transcriptional control. Figure 1 provides a structural overview of HPV.^[28]^

**Table 1:** Human papillomavirus (HPV) E-proteins and functions.

Viral protein	Functions
E1	Viral DNA replication
E2	Viral DNA replication, transcription, and apoptosis
E4	Viral DNA replication, and disruption of cell growth
E5	Immune recognition, modulation, and transformation
E6	p53* and BAK† binding and disruption, cell cycle alteration
E7	Transformation, and disruption of retinoblastoma (pRb)

* Tumor protein P53.

† Bcl-2 homologous antagonist/killer.

### 3.2. Cellular Invasion

The epithelium is an organism first line of defense against invading microorganisms. Micro-abrasions and disruption of this epithelium provide an entry point for papillomaviruses such as HPV. HPV can take advantage of this disruption of the epithelium and directly invade the basal epithelial cells bypassing the now disrupted superficial cell layers. The wound state has been hypothesized to provide a viable ecosystem for HPV to thrive. HPV most likely hijacks soluble factors such as annexin A2, epidermal growth factors, and furin for its own virulent purposes. Interactions between HPV and heparan sulfate proteoglycan (HSPG) has been implicated in the initial point of cell invasion.^[29]^ The expression of HSPG increases in response to the micro-abrasion and is most prevalent at the wound edge.^[30]^ HPV particles attach to the extracellular domain of syndecan-1s (a type I transmembrane proteoglycan) extracellular domain. Syndecan-1 adjoins the extracellular membrane to the cytoskeleton of keratinocytes.^[31,32]^ Secondary ionic interactions between the negatively-charged heparin sulfate molecules on HSPGs and positively-charged L1 motifs on HPVs capsid mediate cellular invasion of the virus.^[33]^ Most HPV types that have been studied enter the cell via clathrin-dependent endocytosis. HPV capsid proteins (L1 and L2) are highly resilient and stable structures with the goal of protecting the encapsulated viral genome. Thus, after initial cellular invasion, conformational changes occur in the L2 capsid protein, allowing for subsequent trafficking to the invaded cell nucleus.^[34]^

**Figure 2.**
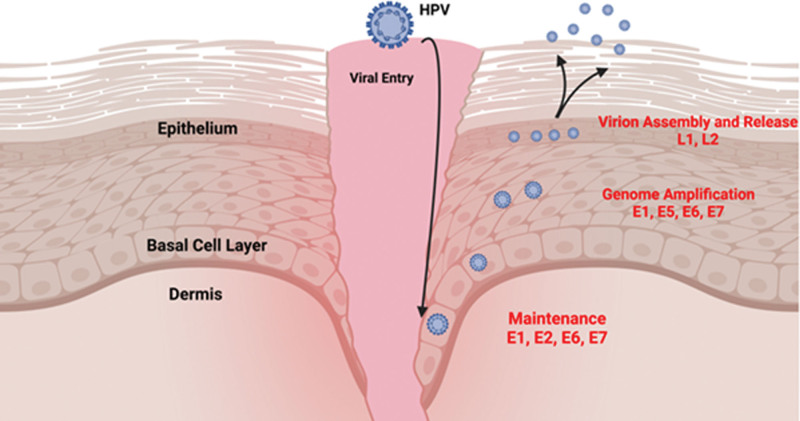
Viral entry occurs secondary to the micro-abrasion and direct invasion of basal epithelial cells. Maintenance phase of HPV occurs in the basal epithelial cells with subsequent migration superficially. Superficial layers of epithelium allow for genome amplification, virion assembly, and subsequent release. Created with BioRender.com (Agreement number: VH24TGERQQ).

The described invasion of the basal epithelial cell by HPV is a strategic one. Basal epithelial cells divide symmetrically and asymmetrically. The symmetric division of basal epithelial cells allows horizontal replication to produce more of them. Asymmetric division refers to the vertical production of daughter epithelial cells. These daughter cells successively move superiorly towards the superficial epithelium acquiring specialized properties associated with tissue regeneration. HPV exploits these cellular mechanisms for its own replication and propagation.^[35]^ Figure 2 provides a general mechanistic overview of the invasion of basal epithelial cells by HPV.

### 3.3. Viral genome integration and oncogenesis

Upon entry into the cell nucleus, HPV begins to exert integrative mechanisms to incorporate itself into cellular DNA. HPV RNA encoding the E6 and E7 oncogenes is initiated via the viral upstream regulatory region. The E6 oncoprotein will bind to p53 and disable its normal function for tumor suppression.^[36]^ This affects the host cell ability to undergo programmed cell death and growth arrest. This inactivation of p53 forces the host cell to enter the S phase of the cell cycle. The E7 oncoprotein will bind to the retinoblastoma protein, causing the release of the transcription factor E2F.^[9]^ The release of E2F activates cyclin-dependent kinases. Subsequent expression of the E1 and E2 oncogenes allows genomic instability at the integration locus. Persistence of the S phase of the cell cycle leads to uninhibited proliferation and differentiation of infected cells, eventually leading to dysplasia.^[37]^

**Figure 3.**
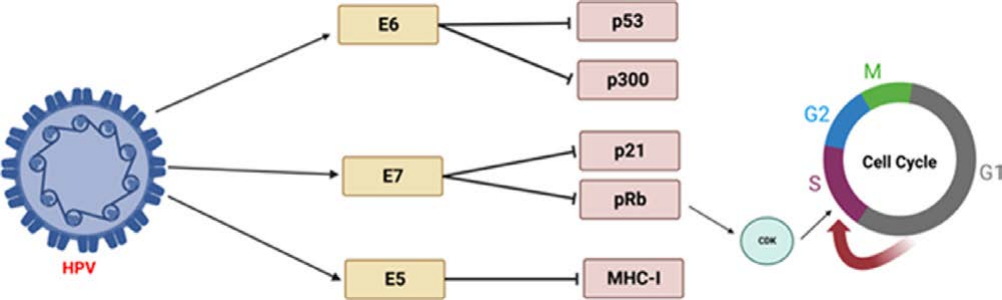
Overview of major HPV oncogenic proteins and their subsequent molecular interactions. E6 protein inhibits both p53 and p300 arresting apoptosis and other downstream mechanisms of programmed cell death. E7 protein inhibits p21 and retinoblastoma protein (pRb) leading to elevated levels of cyclin-dependent kinase (CDK) promoting the S phase of the cell cycle and cellular immortalization. E5 protein inhibits MHC-I protein. Created with BioRender.com (Agreement number: JL24TGETOS).

Although a vast majority of women are inoculated with HPV, many remain asymptomatic. This is due to a rapid and robust response by the immune system to target and destroy infected cells. Thus, in those with a symptomatic infection, evasion or compromise of the immune system is likely occurring. Cancer itself has been described as a disease process mediated by genomic instability.^[38]^ Thus, the consequence of HPV genome integration facilitates the disruption of mitotic checkpoints and progression toward malignancy. Figure 3 provides an overview of HPV oncoproteins and their cellular interactions.^[39]^

## 4. Benign cutaneous lesions

Benign lesions caused by HPV infection, more frequently low-risk genotypes, include verruca vulgaris, plantar warts, flat warts, genital warts, lesions of the head or neck (laryngeal papillomas) or the oral mucosa (verruca vulgaris, condyloma accuminata or disseminated oral papillomatosis). In addition, HPV is involved in the etiology of epidermodysplasia verruciformis, a benign hereditary illness that can become malignant after sun exposure. Immunosuppressed individuals may experience more extensive HPV-related lesions and more recurrent neoplastic alterations.^[40,41]^

### 4.1. Genital lesions

The most prevalent benign genital lesions caused by HPV and the most common sexually transmitted infection are genital warts, also known as condyloma accuminata (CA).^[42]^ Even though the primary cause of genital lesions is a sexually transmitted HPV infection, possible alternative ways of transmission, like vertical or autoinoculation, have been discussed in the literature.^[40]^ Younger ages at the onset of sexual life, an increased number of sexual partners, and a larger number of sex partners of these men are all risk factors for HPV infection in women.^[42]^ In male patients, in addition to the risk factors listed for women, we can also add the absence of circumcision, the nonuse of a condom, and smoking.^[43]^

During sexual intercourse, microscopic injuries to the skin and mucosa of the genital area can occur, allowing viral particles to penetrate the tissue and reach the cells in the basal layer (keratinocytes). The immune system does not fight the viral particles on their way to the basal keratinocytes, a process that is not fully understood.^[44]^ According to some research, HPV suppresses interferon and cytokines activity, preventing inflammation and viremia. Viral replication is not very active at the start of the infection. This one increase and virions are released at the level of the superficial layer when the cells of the basal layer begin to differentiate and reach the spinous and granulosum layers.^[45,46]^

Low-risk genotypes of HPV, particularly types 6 and 11, are involved in the etiology of genital warts. The E6 and E7 proteins of these viral stains reproduce as an episome, the viral DNA conducts independent viral multiplication, and it rarely integrates its genetic sequences into the host DNA.^[47]^

HPV infection can manifest in 3 ways: clinically, subclinically, and latently. The time between first contact with the virus and clinical manifestations can range from 3 weeks to 8 months.^[48]^ There may also be people who never develop visible clinical lesions. Subclinical forms account for 40% of cases in which individuals do not develop clinically visible lesions, but HPV can be detected using paraclinical investigations.^[49]^ Clinically, the lesions begin as papules or nodules with a keratotic surface and then grow in size and number as they confluence to form cauliflower-like lesions. Most frequently, these lesions are asymptomatic, but they can associate itching or bleeding in case of trauma. In 30% of cases, spontaneous regression of the lesions may occur in the first months, regression associated with an increased level of CD4 + lymphocytes in the skin.^[40,50]^

### 4.2. Extragenital lesions

Verruca vulgaris, plantar warts, and flat warts are the most common extragenital manifestations of HPV infection.^[50]^ HPV viral particles can persist on surfaces for several years. As a result, the virus is transmitted through direct or indirect contact of the micro-traumatized skin with viral particles. The mechanism of infection is similar to the one described in genital lesions. After the viral particles bind with basal keratinocyte receptor molecules, the viral DNA begins to replicate independently of the host genome using E1 and E2 proteins, evading the immune response. Following that, skin cells continue to differentiate normally, and viral protein development is enhanced with the assistance of E5 proteins, which improve signaling from grow factor. Protein E2 recruits viral DNA strands to the host nucleus, where the DNA is organized into capsids made up of L1 and L2 proteins, which are expressed by the surface cells. Finally, the viral particles are released during the normal skin desquamation process, which explains the clinically hyperkeratinized lesions.^[37,51]^

Verruca vulgaris is more commonly caused by HPV types 2 and 4, and less frequently by type 49. They are asymptomatic keratotic papules found on the fingers or the dorsal area of the hand, but they can also develop in the periungual region. HPV types 1, 2, and 4 cause plantar warts. They appear clinically as papules or plaques with keratotic surfaces and central black dots on the feet, particularly in high-pressure areas. Flat warts, also known as verruca plana, are linked to HPV 3, 10, 28, and 49. Clinically, they appear as flat skin-colored or gray papules on the face, hands, or legs and can be associated with the Koebner phenomenon.^[41,51]^

Laryngeal papillomatosis is one of the most common benign mesenchymal lesions of the larynx that affects both children and adults, with a high tendency of recurrence after treatment. HPV types 6 and 11, which are frequently found in CA, are implicated in its etiology. Uncertainty surrounds the disease pathogenesis and transmission method. However, it is believed that in cases involving children, vertical transmission from a mother who has just developed genital warts is responsible. In adults, oral sex is thought to be a factor. Regarding pathophysiology, it is thought that viral particles penetrate the basal cells of the squamous mucosa, activate the growth factor, and suppress T cell response leading to DNA replications. Clinically, this condition is represented by an exophytic lesion that might affect breathing and swallowing and require repeated surgery due to recurrence.^[51]^

Direct or oral-genital sexual contact can cause oral manifestations of HPV infection, with HPV types 13, 32, and 57 being the most common. Verruca vulgaris is an exophytic lesion that can appear on the gingiva, labial mucosa, or tongue and is caused primarily by HPV 2 and 57.

Condyloma accuminata is characterized by a cauliflower-like appearance of numerous pale elevated lesions caused mainly by types 6 and 11. Heck disease, also called focal epithelial hyperplasia, presents as a gray or white papule or plaque that occur often on the oral mucosa of children and can disappear on its own.^[52]^

## 5. In situ squamous cell carcinoma

### 5.1. Bowenoid papulosis

Bowenoid papulosis (BP) is a rare condition, part of the clinical spectrum of anogenital intra-epithelial neoplasia, caused by HPV, usually high-risk subtypes 16 and 18, but other variants were also reported. The risk factors overlap with those for HPV infection: early sexual contact, multiple sexual partners, uncircumcised male sexual partners, men who have sex with men, and immunosuppression.

Kopf and Bart first described this condition in 1977, and in the present, some authors suggested the use of penile/vulvar intraepithelial neoplasia or squamous intraepithelial lesion.^[53,54]^

BP is an uncommon, precancerous condition that typically affects young, sexually active adults (mean age around 30), with an equal male-to-female ratio.^[55]^ HPV types 16 and 18 were frequently detected in BP lesions (using PCR for the detection of HPV DNA), but other variants were also reported—6, 11, 18, 31, 33, 34, 39, 51, 52, 53.^[56,57]^

Clinically, it presents as asymptomatic or slightly pruritic, multiple, smooth or velvety, flat-topped, skin-colored, red, violaceous, brown or black papules or plaques. These lesions develop over a short period of time. The most common site of involvement is the anogenital region and thighs, but there are reports of rare extragenital cases, in the head and neck (also involving the oral cavity), with or without associated genital lesions.^[58,59]^ In men, the predilection sites are the shaft of the penis, glans, foreskin and scrotum, while in women, unilateral or bilateral lesions involving labia minor, major or vulva can be seen. Some cases of extragenital BP were reported in immunocompromised patients.^[60]^

Classically genital BP can be mistaken for condylomata accuminata, lesions of lichen planus or angiokeratomas.^[59]^ In those cases, a biopsy will elucidate the diagnosis. The main concern is differentiating between BP, Bowen disease and erythroplasia of Queyrat. Unlike the latter 2 entities, which happen at an older age, BP affects younger patients and usually presents as multiple skin-colored or brown papules, in comparison with a unique erythematous plaque, with scaling, characteristic of Bowen disease. The topical application of 5% acetic acid is helpful for the clinician, making subclinical lesions visible.^[61]^

A BP biopsy will show acanthosis, hyper- or parakeratosis, with or without epithelial vacuolation, dyskeratosis, intact basement membrane and atypical mitoses. The distinction between BP and Bowen disease is made by clinicopathological correlation because histologic differentiation is sometimes impossible. The degree of cellular atypia and the follicular involvement help distinguish BP from Bowen disease. Immunohistochemistry for p16 shows diffuse staining of full-thickness epidermis.^[53,57]^

The natural history of BP is not fully understood. It can regress spontaneously, after a long course, persist, recur or progress into invasive squamous cell carcinoma, mainly in immunosuppressed individuals.^[53,57,62]^ Lesions are highly contagious, therefore monitoring the sexual partners is crucial.

Due to the multifocal distribution and also the typical genital localization, classic surgical excision could lead to disfiguring final results. Mohs surgery could be an option. Other recommended treatment options are cryotherapy, electrocoagulation, CO2 laser therapy, and photodynamic therapy. Topical treatment includes 5-fluorouracil, imiquimod, podophyllin, cidofovir, sinecatechin, and retinoids.^[53,61]^ Lucker et al suggested that imiquimod efficacy varies depending on the status of HPV type 16 in the affected cells of patients with BP. Using fluorescent in situ hybridization (FISH), they concluded that patients with episomal HPV type 16 responded better to topical imiquimod than those with integrated HPV type 16.^[63]^

### 5.2. Bowen disease

Bowen disease (BD) is a slow-growing variant of in situ squamous cell carcinoma (SCCis), usually diagnosed in elderly patients, which was first described in 1912 by JT Bowen. After a long course of progression, a minority of cases—3% to 5%, transform into an invasive or metastatic form of SCC.^[61,64]^

Patients diagnosed with Bowen disease are typically elderly, over 60 years, and it is rare in individuals younger than 30 years, except the immunosuppressed patients, which have a higher risk of developing BD. Bowen disease is a multifactorial entity, the major factors being chronic UV exposure, immunosuppression (after organ transplantation, HIV + patients) and Human Papilloma Virus (HPV) infections. Other etiological factors reported are radiotherapy, arsenic exposure, chronic injury or chronic dermatoses. There were rare cases of BD reported in a porokeratosis of Mibelli lesion, in a scar following the smallpox vaccine and also in erythema ab igne lesions.^[65–67]^

Regarding HPV infection, a wide variety of HPV strains were detected in Bowen disease, but high-risk types were much more identified.^[68]^ Neagu et al analyzed 290 cases of Bowen disease, and the majority of HPV-involved variants were beta (55,84%) and alpha (38,96%), with only 5,19% gamma type.^[69]^ Conforti et al suggested that beta-HPV variants only have an initial role in carcinogenesis, not in the perpetuation phase, supporting the so-called “hit-and-run” theory.^[70]^ The subtypes of HPV involved in Bowen disease are 16, 18, 31, 33, 54, 56, 58, 61, 62, and 73 in situ hybridization analysis of BD affecting the nail showed the association with HPV types 16, 31, 33, 56 and 71. In BD affecting the vulvar area, a strong link with HPV 16 has been described.^[66]^

Other risk factors were described in the literature, such as Caucasian race, the majority of patients have phototypes I and II; Merkel cell polyomavirus and primary Sjogren syndrome have been associated with Bowen disease but without a clear cause and effect assumption.^[65]^

Cases of BD affecting the genitalia and nails are more likely to be linked with HPV infection.^[71]^

The classic clinical presentation is a unique (but could be more than one lesion in 10% to 20% of patients) pink/salmon/erythematous, well-demarcated patch or plaque, with fine yellow/white, easily detached scale or hyperkeratosis on the surface.^[66,72]^

The localization is usually on sun-exposed areas (head, neck, legs), but can affect any skin area (trunk, genital region, nails, palms and soles).^[65]^ While in the classic presentation, the lesion is not associated with any symptoms, in larger lesions, some patients may complain of pruritus. Bleeding, ulceration, and nodule formation on the surface of a typical erythematous BD lesion suggest the progression to invasive SCC.^[66]^

Erythroplasia of Queyrat (EQ) represents a particular form of in situ SCC located on the mucosa of the penis. It is characterized by a velvety erythematous lesion. It can also affect the urethra. EQ showed the presence of high-risk HPV strains such as 16 or 18. Less common variants may clinically present in form of a pigmented plaque, discolored patch or plaque of the genitalia or nail dystrophy.^[72]^

As in BP, BD specimen biopsy shows hyperkeratosis, parakeratosis and acanthosis, with full-epidermal thickness keratinocyte atypia and intact basement membrane. Secondary amyloid deposition might suggest regression of the tumor.^[66]^ The main histological difference between BP and BD is the ratio of cytologic atypia and the absence or minimum count of plasma cells in BP.^[58]^ In some cases, when the scaling is severe, the lesion could easily be mistaken for psoriasis. The unicity of the lesion will help the clinician diagnosing BD.^[66]^ Other differentials of BD include actinic keratoses, discoid eczema, superficial BCC, lichen planus and lichen simplex.^[72]^ Metalloproteinases are responsible for the development of invasive SCC, by destroying the basement membrane. Transformation in invasive SCC is more common in elderly and immunosuppressed patients. The production of Tat protein and the decreased expression of E6 and E7 proteins in HIV-positive patients are the main mechanisms of progression into invasive SCC.^[66]^

## 6. Squamous cell carcinoma

cSCC, developing from epidermal keratinocytes, is the second most prevalent form of skin cancer, following BCC.^[73]^ The development of SCC is a gradual process as a consequence of mutations of the genes involved in the homeostasis of epidermal cells influenced by external factors. cSCC is typically treated surgically, but some patients experience metastases or recurrence, which makes the disease a severe health problem.^[74]^

Chronic exposure to UVR, particularly in the context of field cancerization determined by cumulative sun exposure, is the most important risk factor involved in the etiopathogenesis of cSCC. Certain genetic syndromes, including xeroderma pigmentosum and albinism or immunosuppression, which increases the chance of developing multiple, aggressive cSCC, are additional risk factors that predispose to the development of cSCC.^[75]^

It is considered that HPV may have a role in the development of cSCC. Among the 5 subtypes of HPV, Alpha, Beta (b), Gamma, Mu, and Nu human papillomavirus, Beta-HPV is the most frequently detected in carcinogenesis processes. Beta-HPV may play a role in the etiopathogenesis of cSCC in 2 categories of patients. First, in patients with immunosuppressed conditions, including solid organ transplant recipients and HIV-positive individuals.^[76]^ The second category of patients includes those with epidermodysplasia verruciformis (EV), an autosomal recessive skin disease that Lewandowsky and Lutz first described in 1922. This condition is caused in about 90% of cases by Beta-HPV 5 and Beta-HPV 8 types.^[13]^ Epidermodysplasia verruciformis is characterized by persistent HPV infection, caused by a decrease in the immune system capacity to eradicate HPV infection. This is followed by the development of verruciform lesions that progress toward cSCC in a proportion of 30 to 70% of patients, mainly in cutaneous areas that are exposed to UVR.^[14,77]^ In the general population, HPV is rarely involved in the development of cSCC, the viral load is low, and the keratinocyte genotoxic effect caused by UV radiation is adequately suppressed in immunocompetent patients.^[78]^

Regardless of the immune status of the patients, it is considered that HPV has a role in the etiology of premalignant and malignant lesions. The molecular pathways involving beta-HPV in the carcinogenesis of cSCC are currently being investigated. Not through the direct carcinogenic effect of viral strains, but rather through the loss of T cell immunity, HPV favors the early onset of cSCC in immunosuppressed patients. The repair of viral DNA and cellular apoptosis that are required after the immediate impact of UV on the keratinocyte cell is therefore inhibited by persistent HPV infection in immunosuppressed patients, thereby promoting the development of malignancy.^[79]^ Moreover, UVR can suppress cutaneous cell-mediated immune responses, which promotes the local proliferation of HPV strains.^[80]^ The pathogenesis of cSCC is characterized by a “hit-and-run” mechanism in which HPV is involved in the initiation of the oncogenic process rather than in its progression.^[81]^

There are multiple mechanisms by which HPV influences the development of cSCC in a synergistic manner with UVR exposure. The beta-HPV38 E6 and E7 proteins were found to increase the susceptibility of keratinocytes to UVR in transgenic mice.^[82]^

The E6 oncoprotein of B-HPV type 5 and 8 inhibits SMAD3 transactivation by destabilizing the SMAD3/SMAD4 complex and decreases the transforming growth factor β (TGFβ) signaling pathway. In addition, this molecule typically determines the synthesis of CDK (cyclin-dependent kinase) inhibitors with a role in the cell cycle. The progression from the G1 phase to the S phase of the cell cycle is encouraged by the degradation of SMAD3, a key component of the transforming growth factor-beta 1 (TGF-beta1) signaling pathway, which can promote uncontrolled replication of viral DNA and even cell transformation.^[83,84]^

Beta HPV-5 and Beta HPV-8 E6 oncoproteins can directly interact with Mastermind-like transcriptional coactivator 1 (MAML1), inhibiting Notch signaling in keratinocytes. The Notch 1 pathway plays an important role in cell differentiation and proliferation, and the loss of keratinocyte Notch 1 favors the development of cSCC.^[77]^ The RAS pathway directly involved in developing squamous cell carcinomas in human keratinocytes is stimulated by inhibiting the Notch pathway.

Another mechanism involved in carcinogenesis caused by beta-HPV highlights that b-HPV strains 5, 8, 20, and 38 through proteins E2,6,7 determine the invasion of slow-cycling epidermal stem cells, found, for example, in the hair follicle bulge, thus enabling the persistence and accumulation of DNA damage necessary to generate malignant stem cells.^[85]^ The possibility of developing strategies to reduce the incidence of cSCC in high-risk patients with verruciform epidermodysplasia and immunosuppressed patients relies on the hypothesis of HPV involvement in carcinogenesis. This could be achieved by developing beta-HPV vaccines and studying other co-risk factors in these patients to better understand the molecular mechanisms underlying the development of cSCC.^[77]^ According to Johnson L. et al, the increased seropositivity of beta-HPV in immunocompromised patients with a high risk of developing SCC can be explained by the depletion of CD8 + type T lymphocytes. This highlights the importance of a vaccination strategy that promotes anti-HPV immunity in preventing tumorigenesis.^[81]^

## 7. Other skin cancers related to HPV

Although the oncogenic role of HPV strains is well known, and currently, multiple studies have highlighted the presence of beta-HPV in BCC and melanoma biopsies, it is difficult to establish a molecular mechanism by which the virus is involved in these processes. Moreover, we cannot establish whether the presence of HPV is a factor that initiates the process of oncogenesis or, on the contrary, is a result of changes in local immunity caused by tumor proliferation or even a “contamination” of the surface of the biopsies.^[17,86]^ Regarding the pathogenesis of BCC, one of the proposed mechanisms is the alteration by the virus of the defense mechanisms against the effects of UVR; which promotes carcinogenesis in the photo-exposed areas, which are predilected for the appearance of BCC (the E6 protein of HPV inhibits the action of the pro-apoptotic Bak protein).^[87]^ Another suggested mechanism is the upregulation of p16INK4a and Akt2 expression by beta-HPV 2, possibly through the E7 protein, indicating either a direct role in the carcinogenesis of some BCC subtypes or a marker of active viral infection.^[88]^

In the case of the role of HPV infection in the occurrence and progression of melanoma, the epidemiological data presented in Section 2 reveal the identification of some HPV strains in melanoma biopsies. The incriminating mechanisms are not elucidated, but it was observed on in vitro specimens that E6 and E7 proteins have similar roles in melanocytes and keratinocytes; these are represented by the inhibition of the p53 protein (with a role in DNA repair), respectively by the early activation of cyclins A and E, which leads to changes in the cell cycle.^[19]^ However, as in cSCC and BCC, the oncogenic role of HPV is not established, not knowing if it is an initiator or just a cofactor of UV-induced oncogenesis.^[19]^

## 8. Conclusions

In conclusion, the involvement of HPV in the development of skin cancers requires further investigation both in terms of molecular studies, as well as comprehensive assessment of patient cohorts.

## Acknowledgments

The authors would like to thank Prof Baki Akgül, Institute of Virology, Medical Faculty and University Hospital Cologne, University of Cologne, Cologne, Germany, for his constructive criticism and suggestions for improvement of the manuscript.

## Author contributions

**Conceptualization:** Elena-Codruta Cozma, Laura Mădălina Banciu, Ana Maria Celarel, Bahadar S. Srichawla, Elena Soare, Vincent Kipkorir, Mihnea-Alexandru Găman.

**Investigation:** Ana Maria Celarel, Mihnea-Alexandru Găman.

**Methodology:** Elena-Codruta Cozma, Laura Mădălina Banciu, Ana Maria Celarel, Bahadar S. Srichawla, Elena Soare, Mihnea-Alexandru Găman.

**Project administration:** Laura Mădălina Banciu, Ana Maria Celarel, Elena Soare.

**Resources:** Laura Mădălina Banciu, Mihnea-Alexandru Găman.

**Software:** Laura Mădălina Banciu, Ana Maria Celarel, Bahadar S. Srichawla.

**Supervision:** Elena-Codruta Cozma.

**Validation:** Elena-Codruta Cozma.

**Visualization:** Elena-Codruta Cozma, Laura Mădălina Banciu, Ana Maria Celarel, Bahadar S. Srichawla, Elena Soare, Mihnea-Alexandru Găman.

**Writing – original draft:** Elena-Codruta Cozma, Laura Mădălina Banciu, Ana Maria Celarel, Bahadar S. Srichawla, Elena Soare, Vincent Kipkorir, Mihnea-Alexandru Găman.

**Writing – review & editing:** Elena-Codruta Cozma, Laura Mădălina Banciu, Ana Maria Celarel, Bahadar S. Srichawla, Elena Soare, Vincent Kipkorir, Mihnea-Alexandru Găman.
